# No association between vitamin D status and COVID-19 infection in São Paulo, Brazil

**DOI:** 10.20945/2359-3997000000343

**Published:** 2021-03-19

**Authors:** Cynthia M. Álvares Brandão, Maria Izabel Chiamolera, Rosa Paula Mello Biscolla, José Viana Lima, Cláudia M De Francischi Ferrer, Wesley Heleno Prieto, Pedro de Sá Tavares Russo, José de Sá, Carolina dos Santos Lazari, Celso Francisco H. Granato, José Gilberto H Vieira

**Affiliations:** 1 Grupo Fleury São Paulo SP Brasil Grupo Fleury, São Paulo, SP, Brasil

**Keywords:** Vitamin D, coronavirus, COVID-19

## Abstract

In recent years the immunomodulatory actions of vitamin D, a steroid hormone, have been extensively studied. In 2020, due to the COVID-19 pandemic, the question arose as to 25(OH)D status would be related to susceptibility to SARS-CoV-2 infection, since several studies pointed out a higher prevalence and severity of the disease in populations with low levels of 25(OH)D. Thus, we investigated the 25(OH)D levels in adults “Detected” positive for SARS CoV-2 by RT-PCR (reverse transcriptase polymerase chain reaction) test, and in negative controls, “not Detected”, using the Fleury Group's examination database, in Sao Paulo, Brazil. Of a total of 14.692 people with recent assessments of 25(OH)D and RT-PCR tests for COVID-19, 2.345 were positive and 11.585 were negative for the infection. The groups did not differ in the percentage of men and women, or in the age distribution. There were no differences in the distribution of 25(OH)D between the two groups (p = 0.08); mean 25(OH)D of 28.8 ± 21.4 ng/mL and 29.6 ± 18.1 ng/mL, respectively. In the specific population studied, clinical, environmental, socioeconomic and cultural factors should have greater relevance than 25(OH)D in determining the susceptibility to COVID-19.

## INTRODUCTION

The musculoskeletal effects of vitamin D are widely studied, as well as its endocrine actions in regulating the homeostasis of calcium and phosphorus. Cholecalciferol or “vitamin D”, synthesized in the skin, is metabolized in the liver to 25-hydroxycholecalciferol (25(OH)D) and then in the kidney to its biologically active form, 1,25-dihydroxycholecalciferol (1,25(OH)2D). The metabolite 25(OH)D is the major circulating form of vitamin D in humans, and it is used to reflect person's vitamin D status.

Vitamin D deficiency causes secondary hyperparathyroidism, osteomalacia, osteopenia and an increased risk of fractures. In addition to its classic functions in osteomineral metabolism, the extensive distribution of vitamin D (VDR) receptors in human tissues and the action of the active hormone, 1,25(OH)2D (calcitriol), in regulating the transcription and expression of countless genes, indicate the importance of nonskeletal actions of this hormone. Experimental and clinical studies have revealed the intracrine action of vitamin D in the immune system, particularly in monocytes and macrophages, with a modulating role for both innate and adaptive immune responses against a number of microorganisms, including viruses (1,2).

Autophagic encapsulation of viral particles is also a cellular process enhanced by both 25(OH)D and 1,25(OH)2D (3), with a fundamental role in reducing viral infectivity, for example for human immunodeficiency virus type 1 (4). In addition, other data revealed a role of vitamin D in pulmonary protection against acute respiratory infections, in particular its action on capillary permeability, which plays a fundamental role in the pathophysiology of many diseases with pulmonary involvement (5,6). Respiratory epithelial cells constitutively express 1α-hydroxylase resulting in local activation of vitamin D. Vitamin D-dependent genes including cathelicidin and CD14 are upregulated after the exposure of airway epithelial cells to the inactive vitamin D precursor (7).

In 2020, during the coronavirus disease (COVID-19) pandemic, several retrospective studies were published, showing an association between low 25(OH)D status and increased susceptibility to and severity of SARS-CoV-2 infection, suggesting a deleterious effect of hypovitaminosis D on the incidence and clinical evolution of COVID-19 (8–12). Experimental research has shown that 1,25(OH)2D modulates the expression of angiotensin-converting enzyme 2, which is the receptor for the entry of SARS CoV-2 into cells. VDR-null mice showed more severe acute lung injury in a sepsis model than their wild-type counterparts (13). Thus, it became essential to study the relationship between 25(OH)D and SARS CoV-2 incidence, with the aim of identifying an easily modifiable factor that can play a preventive role in all populations susceptible to infection.

## OBJECTIVE

To compare 25(OH)D levels between individuals infected with SARS-CoV-2, with diagnostic confirmation by RT-PCR (reverse-transcriptase polymerase chain reaction), and individuals negative for SARS-CoV-2, using the Fleury Group's database.

## SUBJECTS AND METHODS

### Data source

Fleury Group is a medical organization that provides supplemental health services in Brazil. Data were collected from the Fleury Group's Caché database, of 14692 individuals who underwent RT-PCR tests for the diagnosis of COVID-19, from March to July 2020, who also had 25(OH)D measured; participants were identified by a unique register number. The study protocol was approved by the research and ethics committee of Fleury Group (protocol number 4.409.445, CAAE 39961120.7.00005474). Informed consent was not required since the data were anonymized.

### Study design

This was a retrospective study that collected records from individuals of both genders, between 18 and 90 years old, with RT-PCR results for SARS CoV-2 and who simultaneously had their 25(OH)D measured over a period of 30 days before or after the collection of the sample for COVID-19 RT-PCR test. In cases of patients with more than one vitamin D test, the most recent in relation to the RT-PCR date, was selected. Records with 25(OH)D above 100 ng/mL were excluded to avoid distortions in the analysis of vitamin D averages. After removing entries with missing data and inconclusive diagnostic tests for COVID-19, the new dataset (n = 13930) was divided into two groups: “Detected” or positive for COVID-19 (2345 patients) and “Not Detected” or negative for COVID-19 (11585 patients).

### Biochemical analysis

25OH vitamin D – Liason, CLIA, DiaSorin, Saluggia, Italy, reference range: 20-60 ng/mL, intra and inter-assay coefficient of variation are 6.0% and 8.0%, respectively; RT-PCR – molecular test developed entirely in house according to the Charité protocol and a confirmatory test by the CDC protocol when necessary for confirmation, using clinical samples from the respiratory tract.

### Statistical analysis

To assess the significant differences between the groups, the normality of the two groups was confirmed (Kolmogorov-Smirnov Normality Test) and then, the difference between the means of the groups was verified using the Welch T test (Software R, www.r-project.org). This is a parametric test, adapted from the Student's t-test, whose objective is to compare two independent groups, without the hypothesis of equal population variance. The test considers the difference between the number of patients in each group when calculating the real difference between the means (14). Statistical significance was defined as p < 0,05.

## RESULTS

There was no difference between groups regarding the percentage of men and women, or regarding the age distribution. There was no significant difference for the mean 25(OH)D between men and women, or between adults and the elderly (over 60 years), (multiple T tests with Bonferroni correction). The Detected Group had a mean 25(OH)D of 28.8 ± 21.4 ng/mL, with a median of 26.0 ng/mL. The not Detected Group had a mean 25(OH)D of 29.6 ± 18.1 ng/mL, with a median of 27.0 ng/mL. Table 1 shows the percentage of individuals and the means of 25(OH)D, separated by the ranges of the values, < 12 ng/mL, 12-20 ng/mL, 20-30 ng/mL and > 30 ng/mL, in both Detected and not Detected groups (15,16).

**Table 1:** Number of patients, mean and percentage of patients by ranges of 25(OH)D, for the “Detected” and “not Detected” groups. Multiple T tests with Bonferroni correction were applied; t-test were performed using the log-transformed values of the means

25(OH)D	Group	n	%	Means 25(OH)D ng/mL	Group	n	%	Means 25(OH)D ng/mL	p value
< 12 ng/mL	Detected	137	5.84	8.68	Not detected	579	5.0	9.01	0.10
12-20 ng/mL	Detected	448	19.1	15.83	Not detected	2066	17.8	16.08	0.13
20-30 ng/mL	Detected	911	38.8	24.51	Not detected	4306	37.2	24.6	0.42
> 30 ng/mL	Detected	849	36.2	43.4	Not detected	4634	40.0	42.84	0.73

**Figure 1:**
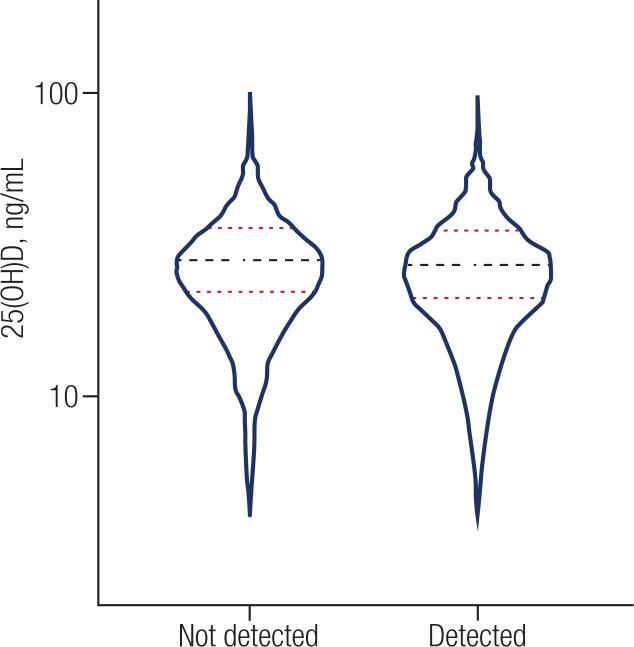
The image shows the dispersion of 25(OH)D, in patients Detected and Not Detected for COVID-19; means are indicated by the black line and two standard deviations (SD) by the red line.

There was no difference in the distribution of 25(OH)D between the “Detected” and “not Detected” groups (p = 0.0811). Figure 1 shows the dispersion of the values of 25(OH)D between the Detected and not Detected groups.

## DISCUSSION AND CONCLUSIONS

Studies prior to the COVID-19 pandemic period have demonstrated the role of vitamin D in innate and adaptive immunity, particularly in protection against viral and bacterial infections. Martineau et al demonstrated, in a large meta-analysis of randomized controlled trials, that vitamin D supplementation reduced the risk of experiencing at least one acute respiratory tract infection (17). The initial spread of COVID-19 occurred in countries that were going through the winter, had a high prevalence of hypovitaminosis D. Together, these data raised the question of the role of vitamin D to susceptibility and the severity of the disease.

Numerous studies initially linked 25(OH)D status to susceptibility and mortality from SARS-CoV-2, although causality cannot be demonstrated (8–12). The clinical evolution and severity of COVID-19 respiratory disease has enormous complexity and competition from numerous other confounding factors, such as obesity, hypertension, socioeconomic level, quality of medical care, comorbidities and probably, the degree of exposure and genetic susceptibility. However, individuals with inadequate 25(OH)D could have an additional risk of contracting a viral infection such as COVID-19, and possibly a greater risk of an unfavorable clinical course (3,6,18). Additionally the social isolation, imposed to control the pandemic, could be a predisposing factor to less sun exposure.

However, our study showed no difference in 25(OH)D status in a large group of Brazilian infected individuals with SARS CoV-2 and non infected controls. The same conclusion was reached by Hastie and cols. (19) and Raisi-Estabragh and cols. (20), using UK Biobank data. Neither study supports the hypothesis of a link between vitamin D levels and the risk of SARS CoV-2 infection, nor does 25(OH)D explain the ethnic differences in COVID-19 prevalence.

The population sample evaluated in this study has a high socioeconomic level, has access to private medical services, and is predominantly of Caucasian origin; therefore, we were unable to assess socioeconomic or ethnic-racial factors that could affect infectivity. Another aspect to be considered is that the pandemic spread in Brazil during late summer and early fall, periods characterized by higher levels of solar irradiation; therefore, low 25(OH)D is less prevalent. Unfortunately, we were also unable to control for other clinical risk parameters for COVID-19, such as weight, diabetes and other comorbidities.

Despite all of the evidence described in the literature on the immunological action of vitamin D, we did not observe differences between 25(OH)D status and COVID-19 susceptibility in a large Brazilian population sample. The strength of this study is the number of participants, mostly Sao Paulo residents, the largest city in Brazil located in the southeastern region of the country. The study population, both with and without SARS CoV-2 infection, has a lower prevalence of hypovitaminosis D, compared to that described in the European or American populations, or even within specific population subgroups living in Sao Paulo, such as the elderly over 80, institutionalized or chronically ill patients (16).

In conclusion, clinical, environmental, socio-economic and cultural factors have greater relevance than vitamin D status in determining the susceptibility to SARS-CoV-2 infections in the population studied.
